# Apport diagnostique de la cervicotomie exploratrice: étude rétrospective de 300 cas

**DOI:** 10.11604/pamj.2015.22.364.8218

**Published:** 2015-12-14

**Authors:** Youssef Darouassi, Mehdi Chihani, Mohamed Mliha Touati, Haddou Ammar, Brahim Bouaity

**Affiliations:** 1 Service d'Oto-rhino-laryngologie, Hôpital Militaire Avicenne, Marrakech, Maroc

**Keywords:** Cou, masses, adénopathies, kystes, cervicotomie, neck, masses, adenopathies, cyst, cervicotomy

## Abstract

Les tuméfactions cervicales représentent un motif fréquent de consultation, et les adénopathies en constituent l’étiologie la plus fréquente. L'examen clinique et les bilans paracliniques permettent, dans la majorité des cas de retrouver une étiologie. Néanmoins certaines de ces tuméfactions restent d'origine non précisée, portant donc l'indication d'une cervicotomie exploratrice. Il s'agit d'une étude rétrospective d'une série de 300 cas de tuméfactions cervicales isolées colligées au service d'ORL de l'hôpital militaire Avicenne de Marrakech entre 2001 et 2014. Tous nos patients ont bénéficié d'une cervicotomie exploratrice avec étude anatomo-pathologique. L’âge des patients varie entre 1 et 76 ans avec un âge moyen de 32,57 ans et une légère prédominance masculine de 52%. La symptomatologie qui a motivé une consultation chez 81% des patients était la tuméfaction latérocervicale. La localisation la plus fréquente était sous mandibulaire (33,34%). Les tuméfactions d'installation progressive ont été retrouvées chez 93,34% des patients. Les principales étiologies retrouvées dans notre étude après examen anatomopathologique étaient de deux types: soit d'origine ganglionnaire dominées par la tuberculose ganglionnaire cervicale (53,66%), le lymphome malin non hodgkinien (6,66%), les adénites réactionnelles non spécifiques (4,66%), la maladie deHodgkin (4,33%) et les métastases ganglionnaires cervicales (3,33%); soit d'origine non ganglionnaire dont le lipome cervicale (17,66%), les kystes branchiaux (6%), les kystes du tractus thyréoglosse (1,66%) et le lymphangiome kystique (1,66%). A la lumière des résultats obtenus et des données de la littérature, nous allons discuter l'intérêt et l'utilité de la cervicotomie exploratrice dans le diagnostic étiologique des tuméfactions cervicales lorsque les examens cliniques et paracliniques ne sont pas concluants, et ainsi d'analyser les aspects épidémiologiques, cliniques et paracliniques des différentes étiologies retrouvées. La cervicotomie exploratrice reste, avec l’étude anatomo-pathologique, un outil nécessaire pour le diagnostic de certitude de certaines tuméfactions cervicales malgré son caractère invasif.

## Introduction

Les tuméfactions cervicales représentent un motif fréquent de consultation, et les adénopathies en constituent la cause la plus fréquente. L'interrogatoire, l'examen clinique en particulier l'examen oto-rhino-laryngologique permettent dans la majorité des cas de retrouver une étiologie, néanmoins certaines de ces tuméfactions restent d'origine non précisée, portant donc l'indication d'une cervicotomie exploratrice qui constitue alors le dernier examen complémentaire et parfois le premier voire l'unique geste thérapeutique. Les tuméfactions cervicales peuvent être d'origine ganglionnaire ou non ganglionnaire, et les grandes causes d'adénopathies sont généralement: infectieuses, hématologiques et métastatiques.

## Méthodes

Notre étude est rétrospective portant sur 300 patients présentant des tuméfactions cervicales isolées et ayant subi une cervicotomie exploratrice, colligées au service d'oto-rhino-laryngologie et de chirurgie cervico-faciale de l'hôpital militaire Avicenne de Marrakech, sur une période de 13 ans, allant de 2001 au 2014. Le but de ce travail est de discuter l'intérêt et l'utilité de la cervicotomie exploratrice dans le diagnostic étiologique des tuméfactions cervicales, lorsque les examens cliniques et paracliniques ne permettent pas d'orientation étiologique précise, et également d'analyser les aspects épidémiologiques, cliniques, et paracliniques des différentes étiologies retrouvées après l’étude histologique.

## Résultats

L’âge de nos patients variait entre 1 et 76 ans, avec un âge moyen de 32,57 ans. Nous avons noté une prédominance masculine dans 52% des cas, avec un sexe ratio de 1,08. Les habitudes toxiques (tabagisme et alcoolisme chroniques) ont été présentes chez 40,34% des patients, et la notion de contage tuberculeux a été notée dans 51,82% des cas. Le délai moyen de diagnostic était de 158,82 jours. La tuméfaction latérocervicale constitue le motif de consultation le plus fréquent dans notre série, présent dans 81% des cas. La localisation la plus fréquente était jugulo-carotidienne dans 33,34% des cas. Les tuméfactions étaient uniques dans 82,66% des cas, mobiles dans 90% des cas, indolores dans 77,66% des cas, la consistance était ferme dans 63,34% des cas, et la peau en regard était normale dans 79,66% des cas. La tuméfaction la plus volumineuse a mesuré 8x7 cm. La radiographie thoracique, la numération formule sanguine, et le bilan endoscopique ont été réalisés chez tous nos patients. Tous les patients ont bénéficié d'une cervicotomie, et l'examen extemporané n'a été réalisé que dans 20% des cas. La tuberculose ganglionnaire cervicale représente 53,66% des étiologies. Les différentes étiologies retrouvées sont résumées dans le Tableau 1.

**Tableau 1:** Répartition des différents diagnostics retrouvés

Diagnostics retrouvés	Nombre	%
Tuberculose ganglionnaire cervicale	161	53,66
Lymphome malin non hodgkinien	20	6,66
Adénites réactionnelles non spécifiques	14	4,66
Maladie de hodgkin	13	4,33
Métastase ganglionnaire cervicale	10	3,33
Maladie de Kimura	1	0,33
Lipome cervical	53	17,66
Kystes branchiaux	18	6
Kystes du tractus thyréoglosse	5	1,66
Lymphangiome kystique	5	1,66

## Discussion

Les tuméfactions cervicales peuvent être soit d'origine ganglionnaire, représentées essentiellement par les causes infectieuses, hématologiques et métastatiques; soit d'origine non ganglionnaire [1, 2]. Dans notre série, la tuberculose ganglionnaire était la cause la plus fréquente représentant 53,66% des cas. L’âge moyen des patients était de 34 ans ce qui est proche des données de la littérature [3, 4]. Nous avons noté une légère prédominance féminine ce qui rejoint les données de Bourekoua [4] alors que Hochedez [3] a décrit plutôt une prédominance masculine. La localisation la plus fréquente des adénopathies dans notre série était jugulo-carotidienne ce qui est similaire aux données de la littérature [5, 6]. La fistulisation des adénopathies à la peau a été observée chez 30% des patients, ce qui est porche des données de la littérature [7]. La lymphocytose est l'anomalie la plus notée dans la numération formule sanguine [3], ceci a été également observé dans notre série. L'intra-dermo-réaction à la tuberculine a été positive dans 96,66% des cas, ce qui est proche de celui décrit dans la série de Bourekoua [4].

Les hémopathies ont représenté 11% des tuméfactions dans notre série, dont le lymphome malin non hodgkinien dans 6,66% des cas et la maladie de Hodgkin dans 4,66% des cas. Parmi l'ensemble des cancers de la tête et du cou, les lymphomes malins non hodgkiniens occupent la seconde position après les carcinomes épidermoïdes [8]. Dans notre série l’âge moyen était de 54,4 ans ce qui rejoint les données de la littérature où l'on note un âge moyen supérieur à cinquante ans [8–10]. Le sexe masculin prédomine dans notre étude ce qui concorde avec la littérature [9, 10]. La localisation la plus dominante des adénopathies chez nos patients était jugulo-carotidienne ce qui est également décrit par Hariga [9]. Le caractère volumineux des adénopathies notamment supérieur à 5 cm est un élément de mauvais pronostic [11], dans notre série la plupart des adénopathies étaient moins volumineuses. Nous avons noté que le lymphome de type B était le plus fréquent, ce qui rejoint les données de la littérature [9, 10]. La maladie de hodgkin représente 10% de l'ensemble des lymphomes et 1% de tous les cancers [12]. La distribution en fonction de l’âge est bimodal tant chez la femme que chez l'homme, ainsi décrit-on classiquement un premier pic entre 20 et 30 ans, et un second après 50 ans [12]. Dans notre série nous avons noté une prédominance masculine ce qui était également décrit dans les séries de Haddani [13] et Kobris [14]. Les adénopathies peuvent être isolées ou disséminées, souvent unilatérales mais toujours asymétriques [12]. La maladie de hodgkin classique scléro-nodulaire représente la forme la plus fréquente des lymphomes hodgkiniens [13, 14], ce qui est le cas dans notre série. La numération formule sanguine, la radiographie thoracique, la tomodensitométrie thoracique et abdomino-pelvienne, l’échographie abdominale et la biopsie ostéomédullaire sont des bilans à demander systématiquement en cas de maladie de Hodgkin ou lymphome malin non hodgkinien et ils rentrent dans le cadre du bilan d'extension [12]. Les cancers des voies aéro-digestives sont très lymphophiles et peuvent donc donner des métastases ganglionnaires cervicales [11]. L’âge moyen de survenue est supérieur à cinquante ans dans la littérature [11, 15], alors qu'il était de 42,6 ans dans notre série. Le sexe masculin prédomine dans notre série ce qui concorde avec la littérature [11, 15]. La localisation la plus fréquente des adénopathies chez nos patients était spinale, alors qu'elle était sous digastrique dans la série de Gehanno [11] et jugulo-carotidienne dans celle de Mekouar [16].

Un examen clinique bien conduit et les bilans paracliniques sont nécessaires pour chercher le cancer primitif [17]. Dans notre série l'examen anatomo-pathologique a suspecté l'origine primitive au niveau du cavum dans 70% des cas. Le lipome cervical représente l’étiologie non ganglionnaire la plus fréquente des tuméfactions cervicale dans notre série. L’âge moyen de nos patients était de 43,24 ans ce qui est comparable aux données de la littérature [16, 18]. Nous avons noté une prédominance masculine ce qui rejoint les données de la littérature [16, 19], cependant Belfaquir [18] a retrouvé dans sa série une répartition égale entre les sexes. Habituellement le lipome se développe au niveau des extrémités et du tronc, la localisation cervicale du lipome ne représente que 13% de l'ensemble des localisations [18, 19]. Les lipomes sont généralement de petite taille, et dans presque 80% des cas sont de taille inférieure à 5 cm, toutefois des lipomes géants ont été décrits dans la littérature [19, 20]. Le bilan radiologique (échographie, TDM et IRM) pose souvent le diagnostic mais tout aspect hétérogène doit suspecter un liposarcome [18]. Dans notre série d'autres causes non ganglionnaires ont été notées mais elles étaient moins fréquente, notamment le kyste du tractus thyréoglosse, les kystes branchiaux et les lymphangiomes kystiques.

## Conclusion

La cervicotomie exploratrice reste avec l’étude anatomo-pathologique, un examen paraclinique nécessaire pour le diagnostic de certitude des tuméfactions cervicales quand les bilans cliniques et paracliniques ne sont pas concluants, malgré son caractère invasif. Ces tuméfactions peuvent être d'origine ganglionnaire ou non ganglionnaire. Les adénopathies cervicales étaient l’étiologie la plus fréquente des tuméfactions dans notre série, représentées dans 53,66% des cas par la tuberculose ganglionnaire.
